# Design of Multi-Parameter Compound Modulated RF Stealth Anti-Sorting Signals Based on Hyperchaotic Interleaving Feedback

**DOI:** 10.3390/e24091283

**Published:** 2022-09-12

**Authors:** Jinwei Jia, Zhuangzhi Han, Yuying Liang, Limin Liu, Xuetian Wang

**Affiliations:** 1 Shijiazhuang Campus, Army Engineering University, Shijiazhuang 050003, China; 2 School of Aerospace Engineering, Nanchang Institute of Technology, Nanchang 330000, China; 3 School of Information and Electronics, Beijing Institute of Technology, Beijing 100081, China

**Keywords:** radio frequency stealth, anti-sorting, signal design, multi-parameter compound modulation, hyperchaotic system

## Abstract

Radio frequency (RF) stealth anti-sorting technology is a research hotspot in the radar field. In this study, the signal design principles of anti-cluster and anti-SDIF sorting were investigated for processes of clustering pre-sorting and sequence-difference-histogram main sorting. Then, in accordance with the signal design principle, a 2D interleaving feedback hyperchaotic system based on the cosine-exponential was designed. A method to modulate the pulse repetition interval (PRI) of the signal parameters and carrier frequency with wide intervals through the hyperchaotic system was developed. Finally, we verified the correctness of the signal design principle, the performance of the hyperchaotic system, and the anti-sorting performance of the designed signal using simulations. The results showed that the signal design principle could guide the signal design. The hyperchaotic system outperformed the classical 1D and 2D chaotic systems and the classical 3D Lorenz systems in terms of randomness and complexity. Anti-cluster sorting and anti-SDIF sorting could be realized by anti-sorting signals modulated by a hyperchaotic system, with the anti-SDIF sorting performance being better than that of the PRI random jitter signal.

## 1. Introduction

In recent years, with the rapid development of the radio frequency (RF) hardware circuit and signal processing technology, the detection ability of wide-band real-time electronic reconnaissance equipment, as represented by radar warning receivers, has rapidly improved. The real-time lethality of the radar countermeasure system has been greatly enhanced, threatening the survival of radars on the battlefield [1,2]. Therefore, to improve the survival rate of radars on the battlefield, RF stealth radars have become a research hotspot in the radar field. In accordance with the three detection stages of signal interception, sorting, and recognition of broadband real-time electronic reconnaissance equipment, such as radar alarm receivers, the corresponding radar RF stealth strategies mainly include anti-interception [3,4,5], anti-sorting [6], and anti-identification. Anti-interception is primarily achieved through RF stealth, including power control of the radiation source [7,8], directional antenna designs [9], and low-interception waveform designs [10,11]. However, anti-interception technology often enhances radar RF stealth capabilities at the cost of radar performance [12]. Therefore, to further improve the performance of radar detection and RF stealth, anti-sorting signal design technology has become an important method for RF stealth.

Signal sorting is mainly divided into pre-sorting and main sorting processes. Pre-sorting aims to classify the pulses according to the RF, pulse width (PW), and direction-of-arrival (DOA) in the pulse descriptor word (PDW) of the electronic reconnaissance system to realize the dilution of high-density pulse streams. Main sorting is the core of signal sorting for radiation sources. It usually processes the time-of-arrival (TOA) of each pulse to obtain the pulse repetition interval (PRI) and its modulation mode for each radiation source in the electromagnetic environment [13]. Therefore, to weaken the signal sorting of the electronic reconnaissance system, radar mainly addresses the two sorting processes: pre-sorting and main sorting. In engineering practice, the pre-sorting algorithm mainly used is the k-means clustering algorithm based on data field theory, while the main sorting algorithm is generally based on the sequence-difference-histogram algorithm.

Traditional signal design technology for RF stealth anti-sorting is mainly aimed against main sorting based on PRI sorting, including (1) interference pulse anti-sorting [14,15,16,17]—adding an interference pulse signal to the radar signal to disrupt the interception and identification of the PRI information of the pulse signal by the intercepting receiver of enemies; (2) jitter PRI anti-sorting [16,18,19]—adding jitter to the PRI of the pulse signal, where the different PRIs of each pulse signal make it difficult for enemies to intercept the receiver and sort the radar signal; (3) PRI optimization and anti-sorting [20,21]—optimizing the PRI or signal system design, which only requires precise quantitative design of the PRI without additional interference pulse design. Although the above methods have achieved certain results, they lack a comprehensive principle of sorting failure as a theoretical support. Therefore, the stability of the anti-sorting performance of the designed signals needs further verification.

Moreover, as chaos theory has developed, its application has gradually expanded from traditional cryptography and image encryption to other fields. Experts and scholars have attempted to use chaotic systems to modulate the parameters of radar or communication signals. Yang et al. [22] proposed a frequency hopping system based on a 4D hyperchaotic design and modulated the frequency of communication signals using a chaotic system, achieving good RF stealth performance. This result inspired researchers in the belief that chaos and the complex dynamic behavior of hyperchaotic systems have promising applications in signal parameter modulation. Therefore, it is essential to construct a chaotic system that satisfies the real-time requirements of signal modulation and is highly complex. In general, chaotic systems can be divided into 1D and high-dimensional chaotic systems according to the number of variables in the system. A 1D chaotic system has only one variable, while a high-dimensional one has many variables [23,24,25]. A 1D chaotic system has a simple chaotic structure and is easy to realize. However, it is vulnerable to attacks by the group-lifting algorithm due to the limited chaotic interval. Compared with 1D chaotic systems, higher-dimensional ones have stronger unpredictability, complexity, and initial value sensitivity. However, high-dimensional chaotic systems have significant disadvantages, such as extensive computation, complex structures, and difficult implementation. In 1979, Rossler proposed the first hyperchaotic system. Since then, scholars have put forward several hyperchaotic systems successively. Peng [26], Liu [27], and Yang [22] proposed 4D hyperchaotic systems in 2014, 2015, and 2020, respectively. The three hyperchaotic systems were constructed by adding a new state feedback controller based on a 3D chaotic system. During the construction, the newly added controller had to feed back to the original controller, and the original controller in another set had to feed back to the new controller. The feedback allowed the four controllers of the system to interact with each other, making the relationship more complicated. However, chaotic systems are very complicated to implement due to their multiple controllers, and the increase in dimensions leads to heavy consumption of computing resources. Thus, the relationship between the chaotic dimension and randomness needs should be considered comprehensively.

With the development of chaotic systems, researchers discovered the recursive properties of chaotic systems. Recursion was first proposed by Henri Poincare in his seminal work published in 1890 [28]. Although recursion was discovered in nature early on, research on recursive aspects of nature was not well-developed because there was no suitable method to analyze the dynamic model in the high-dimensional phase space at that time. Following the rapid development and application of modern computer technology, Ekman proposed the recurrence plot (RP) in 1987, a powerful tool to study the recurrence characteristics of time series, using 2D graphics for the recurrence analysis of the characteristics of various signals [29]. Additionally, RPs are a new, important tool to deal with recently developed nonlinear and non-stationary problems. Since RPs can only analyze signals qualitatively, Zbilut and Webber et al. proposed recursive quantitative analysis (RQA) to quantitatively analyze various recursive phenomena reflected by signals in the RP and obtain the information needed to study the characteristics of signals [30]. The main purpose of RQA is to characterize the dynamic characteristics of signals through various RQA measures and quantitatively analyze signals. These quantitative analysis methods are mainly intended to count the distribution characteristics of the basic graph points and line segments in the RP, which is based on the density of recursive points, diagonal lines, and vertical lines (horizontal lines) in the RP. After more than 40 years since the emergence of the RP, the existing RQA measures mainly include the recurrence rate (RR), determinism (DET), laminarity (LAM), and the clustering coefficient (CC) of recurrence. These RQA measures play an important role in analyzing non-stationary random signals [31].

In this study, we focused on the theory of anti-sorting signal design, without involving specific hardware implementation. First, the whole process of signal sorting was analyzed, focusing on the principle of failure behind the pre-sorting algorithm, based on data field clustering, and the main sorting algorithm, based on sequential difference histogram (SDIF). Then, based on the failure principle, a multi-parameter composite modulated RF stealth anti-sorting signal was designed using the hyperchaotic system designed in this paper. The anti-sorting performance of the signal was verified through simulations and experiments. The paper is organized as follows. Section 2 analyzes the sorting failure principle for the clustering algorithm and SDIF algorithm in detail. In Section 3, a novel 2D interleaving feedback chaotic system based on cosine-exponential (CE) is proposed, as well as a wide-interval signal design method, and the design of a wide-interval anti-sorting signal based on the CE hyperchaotic system is described. In Section 4, the signal design principle, chaotic mapping performance, and anti-sorting performance of the designed signal are simulated and verified. Section 5 concludes the paper.

## 2. Motivations and Contributions

In the present complex radar electronic countermeasure environment, RF stealth radar has excellent development potential due to its excellent anti-interception and anti-sorting performance. Anti-sorting signal design is an important development direction for RF stealth radar [6,32]. In addition, anti-PRI sorting signal design requires the relevant theoretical basis for the signal design. Therefore, the failure principle of the SDIF sorting algorithm was investigated in this study. Then, the PRIs and RFs of radar signals were designed with the chaotic system according to the failure principle. The signal eventually achieved anti-sorting.

To design a signal with better anti-sorting performance, the failure principle of the clustering sorting algorithm and the SDIF sorting algorithm were studied, providing theoretical support for anti-sorting signal design. The threshold function of the SDIF algorithm will fail when the PRI value of the signals follows an interval distribution with a length of more than 20 times the minimum interval of the PRI, and this failure principle proposed for the first time. Then, in accordance with the failure principle, the PRIs and RFs of radar signals were designed with the chaotic system. As a result, the signal can achieve anti-clustering sorting and anti-SDIF sorting. Simulation results show that the signal has better anti-sorting performance.

## 3. Principle of Anti-Sorting Signal Design

The design principle for the anti-sorting signal is mainly based on research into the signal sorting algorithm aimed at obtaining the sorting failure principle through analysis. The principle of sorting failure provided the theoretical support for the anti-sorting signal design and improved its efficiency and success rates.

### 3.1. Clustering Algorithm and Its Failure Principle

#### 3.1.1. Clustering Sorting Algorithm

For wide-band real-time electronic reconnaissance systems, such as radar warning receivers, the clustering algorithm is usually used in the pre-sorting stage, mainly employing the RF, PW, and DOA in the PDW to classify the pulse and dilute the high-density pulse stream. At present, the k-means clustering algorithm and its improved algorithm are commonly used for clustering sorting. Therefore, this section analyzes the k-means clustering algorithm based on the data field.

The k-means clustering algorithm is a widely used, typical clustering algorithm [33], and its basic principles are as follows.

Assuming that the set $D=\{D_{1},D_{2},D_{3},\cdot \cdot \cdot ,D_{n}\}$ containing N data is the data to be clustered with $D_{j}=\{D_{m1},D_{m2},D_{m3},\cdot \cdot \cdot ,D_{mq}\}$, m=1,2,3,⋅⋅⋅,n. The k-means clustering is used to find a partition interval $P_{k}=\{C_{1},C_{2},C_{3},\cdot \cdot \cdot ,C_{k}\}$ that minimizes the objective function $f\left(P_{k}\right)=\sum_{i=1}^{k}\sum_{D_{l}\in C_{i}}d\left(D_{l},M_{i}\right)$. The objective function expresses the similarity between data, usually taking the Euclidean distance as the objective function. The Euclidean distance equation of $A\left(x_{i1},x_{i2},\cdot \cdot \cdot ,x_{ik}\right)$ and $B\left(x_{j1},x_{j2},\cdot \cdot \cdot ,x_{jk}\right)$ is shown as follows.

(1) $$d\left(i,j\right)={\left(\sum_{k=1}^{n}{\left|x_{ik}-x_{jk}\right|}^{2}\right)}^{\frac{1}{2}}$$

After clustering the data, the central location of category I is:

(2) $$M_{i}=\frac{1}{n_{i}}\sum_{D_{l}\in C_{i}}D_{l}$$

where $n_{i}$ represents the data number in class $C_{i}$. The above is a clustering process. Then, the process is repeated by calculating the new cluster center and adjusting the class. The classification ends when the sum of the squares of the distances from each data point to each center is minimized.

Although the k-means algorithm is classic, it needs to select the number of clusters and the center value of the class in advance, which is not feasible for sorting unknown radar signals. Secondly, there are some abnormal and wrong data in radar signal sorting, and the k-means algorithm is very sensitive to abnormal data. Thus, the k-means clustering algorithm cannot be used directly in radar signal sorting. The data field clustering algorithm can complete the initial clustering without prior knowledge of data, providing the prior knowledge required by the k-means clustering algorithm.

**Figure 1:**
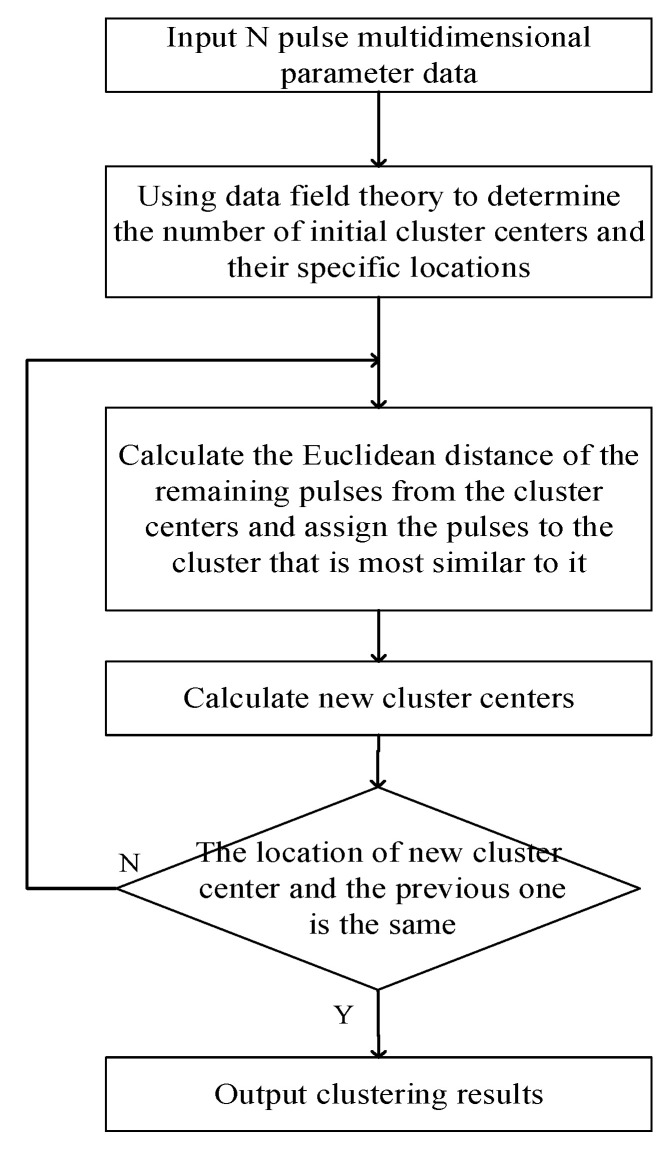
Flow chart of the k-means clustering algorithm based on data field theory.

Therefore, in the practical application of clustering algorithms, data field theory and clustering theory are usually combined. Firstly, the number of data field clusterings is used as the initial clustering number for the k-means algorithm, and then the potential center obtained by data field clustering is used as the initial clustering center of the k-means clustering algorithm. Finally, the k-means clustering algorithm completes the final clustering. The workflow of the algorithm is shown in Figure 1.

#### 3.1.2. Failure Principle of Clustering Sorting

The core step in clustering algorithms is determining the similarity of data points using the Euclidean distance and dividing the initial cluster center using data field theory. Therefore, this paper proposes a joint design method for 3D pseudo-center width agility based on the interval distribution for the Euclidean distance equation and the data field division of initial cluster centers. The method can be explained as follows:

① The 3D parameters refer to the 3D data for the PW, RF, and DOA in the PDW to be sorted by the clustering;
② The interval distribution indicates that the PW, RF, and DOA parameters obey the interval distribution and are no longer fixed or finite fixed values;
③ Wide agility refers to a jump in the data center values of the same dimension in two adjacent intervals to achieve a large jump in interval values;
④ The pseudo-center indicates that the PW, RF, and DOA from the same radiation source are sorted into multiple pseudo-cluster centers in the clustering algorithm after the interval distribution and wide agility design. The clustering algorithm will incorrectly classify the signals from the same radiation source into multiple radiation sources;
⑤ In the joint design, the PW, RF, and DOA signals are simultaneously designed with interval distribution and wide flexibility. The differentiation of the pseudo-cluster centers is made more obvious to prevent the overlap of the pseudo-cluster centers from affecting the anti-sorting performance of the signal.

According to the above analysis, the width agility of 3D parameters is the key to forming pseudo-cluster centers, so studying the jump range of parameters is necessary.

First, the analysis starts with 1D parameters. The center value *x* of the PW signal parameter interval 1 is $x_{10}$, and any value $x\in \left(x_{11},x_{12}\right)$ within the interval is $\Delta_{1}$; the center value *x* of the PW signal parameter interval 2 is $x_{20}$, and any value $x\in \left(x_{21},x_{22}\right)$ within the interval is $\Delta_{2}$. To study the relationship between the amplitude of parameter agility and the interval size, PW values $x_{A}\in \left(x_{11},x_{12}\right)$ and $x_{A}\to x_{12}$ are assumed in the signal PW set.

The Euclidean distance between $x_{A}$ and the center value of interval 1 is:

(3) $$d_{x_{A}x_{10}}=\left|x_{A}-x_{10}\right|$$

The Euclidean distance between $x_{A}$ and the center value of interval 2 is:

(4) $$d_{x_{A}x_{20}}=\left|x_{A}-x_{20}\right|$$

Due to $x_{A}\in \left[x_{11},x_{12}\right]$, $d_{x_{A}x_{10}}<d_{x_{A}x_{20}}$. Therefore,

(5) $$\left|x_{A}-x_{10}\right|<\left|x_{A}-x_{20}\right|$$

Due to $x_{A}\to x_{12}$, Equation (5) can be transformed into:

(6) $$\left|\frac{\Delta_{1}}{2}+x_{10}\right|<\left|\frac{\Delta_{2}}{2}-x_{20}\right|$$

Therefore,

(7) $$x_{20}-x_{10}>\frac{\Delta_{1}}{2}+\frac{\Delta_{2}}{2}$$

According to Equation (7), in the case of 1D parameters, relatively independent multiple pseudo-cluster centers without overlap can be formed when the agility of the central values of two adjacent intervals is at least half of the sum of their intervals. Specifically, if the interval values of two adjacent intervals are equal—i.e., $\Delta_{1}=\Delta_{2}=\Delta$—then:

(8) $$x_{20}-x_{10}>\Delta$$

According to Equation (8), for 1D parameters, if the interval values of two adjacent intervals are equal, the deceleration magnitude of the central value of the adjacent intervals is at least one interval, and relatively independent multiple pseudo-cluster centers without overlap can be formed. The above analysis explores the relationship between the agility range of interval central values and the interval of n-dimensional parameters from the perspective of 1D parameters.

The center value of the n-dimensional parameters of the signal in interval *i* is $\left\{x_{i_{j}0}|j\in \left[1,n\right]\right\}$, and *j* is any dimension of the n-dimensional parameters in the interval. Any value is $\left\{x_{i_{j}}\in \left(x_{i_{j}1},x_{i_{j}2}\right)|j\in \left[1,n\right]\right\}$ in the interval *i*, and the interval length is $\left\{\Delta_{i_{j}}|j\in [1,n]\right\}$. To study the relationship between parameter agility amplitude and interval length, it is assumed that there is a data point A $\left\{x_{1_{j}}\in \left(x_{1_{j}1},x_{1_{j}2}\right)|j\in \left[1,n\right]\right\}$ and $\left\{x_{1_{j}}\to x_{1_{j}2}|j\in \left[1,n\right]\right\}$ in interval 1.

Then, the Euclidean distance between $x_{1_{j}}$ and the center value of interval 1 is:

(9) $$d_{x_{1_{j}}x_{1_{j}0}}={\left(\sum_{j=1}^{n}{\left(x_{1_{j}}-x_{1_{j}0}\right)}^{2}\right)}^{\frac{1}{2}}(j\in \left[1,n\right])$$

The Euclidean distance between $x_{1_{j}}$ and the center value of interval 2 is:

(10) $$d_{x_{1_{j}}x_{2_{j}0}}={\left(\sum_{j=1}^{n}{\left(x_{1_{j}}-x_{2_{j}0}\right)}^{2}\right)}^{\frac{1}{2}}(j\in \left[1,n\right])$$

Due to $\left\{x_{1_{j}}\in \left(x_{1_{j}1},x_{1_{j}2}\right)|j\in \left[1,n\right]\right\}$, $d_{x_{1_{j}}x_{1_{j}0}}<d_{x_{1_{j}}x_{2_{j}0}}$. Thus,

(11) $${\left(\sum_{j=1}^{n}{\left(x_{1_{j}}-x_{1_{j}0}\right)}^{2}\right)}^{\frac{1}{2}}<{\left(\sum_{j=1}^{n}{\left(x_{1_{j}}-x_{2_{j}0}\right)}^{2}\right)}^{\frac{1}{2}}(j\in \left[1,n\right])$$

To ensure that $d_{x_{1_{j}}x_{1_{j}0}}<d_{x_{1_{j}}x_{2_{j}0}}$ is correct continuously,

(12) $${\left(x_{1_{j}}-x_{1_{j}0}\right)}^{2}<{\left(x_{1_{j}}-x_{2_{j}0}\right)}^{2}(j\in \left[1,n\right])$$

According to the geometric meaning of Equation (12), it can be simplified as:

(13) $$\left|x_{1_{j}}-x_{1_{j}0}\right|<\left|x_{1_{j}}-x_{2_{j}0}\right|(j\in \left[1,n\right])$$

Due to $\left\{x_{1_{j}}\to x_{1_{j}2}|j\in \left[1,n\right]\right\}$, Equation (13) can be transformed into:

(14) $$\left|\frac{\Delta_{1_{j}}}{2}+x_{1_{j}0}\right|<\left|\frac{\Delta_{2_{j}}}{2}-x_{2_{j}0}\right|(j\in \left[1,n\right])$$

Therefore,

(15) $$x_{2_{j}0}-x_{1_{j}0}>\frac{\Delta_{1_{j}}}{2}+\frac{\Delta_{2_{j}}}{2}(j\in \left[1,n\right])$$

According to Equation (15), in the case of n-dimensional parameters, when the agility magnitude of the parameter center value of any dimension of two adjacent intervals is at least half of the sum of their intervals under their respective dimensions, relatively independent multiple pseudo-cluster centers without overlap can be formed.

If the interval values of two adjacent intervals are equal—i.e.,

(16) $$\Delta_{1_{j}}=\Delta_{2_{j}}=\Delta$$

then,

(17) $$x_{2_{j}0}-x_{1_{j}0}>\Delta$$

According to Equation (17), for n-dimensional parameters, if the parameter distribution interval values of any dimension of two adjacent intervals are equal, relatively independent multiple pseudo-cluster centers without overlap can be formed when the dexterity magnitude of the parameter center value of any dimension determined by adjacent intervals is at least one interval.

### 3.2. SDIF Algorithm and Its Failure Principle

#### 3.2.1. SDIF Algorithm

Radar signal source sorting, also known as the deinterleaving of the radar radiation source signal, refers to the process of separating radar pulse trains from random and staggered pulse streams. In essence, it consists of the parameter matching of each signal pulse. In engineering, histogram sorting is the most commonly used method to estimate the PRI values of radiation source signals based on the statistical principle. After calculating the difference in the TOAs, the histogram of the difference is formed. Then, an appropriate sorting threshold and strategy are set. The cumulative difference (CDIF) histogram and SDIF are two improved algorithms commonly used in engineering.

Both SDIF and CDIF are TOA difference histogram sorting methods. The two sorting algorithms count the TOA difference of pulses according to certain rules and analyze the PRI estimates. Then, an impulse sequence search is performed based on the PRI estimate. Finally, radiation source pulse trains are extracted [34,35]. Compared with traditional histogram sorting algorithms, SDIF and CDIF algorithms greatly reduce the computational effort, and they work in real-time. The SDIF and CDIF algorithms widely used in engineering can sort fixed PRI, staggered PRI, and jitter radiation source signals [36,37,38].

Compared with the CDIF algorithm, the SDIF algorithm has the following advantages. SDIF only analyzes the histogram of the current level without the histogram statistics of different levels and the 2× PRI test. It requires less computation and has a faster processing speed. In addition, the SDIF algorithm has an optimized threshold function. When combined with sub-harmonic detection, false detection of the SDIF algorithm can be avoided. Therefore, SDIF is more widely used than CDIF. The flows of the SDIF algorithm are as follows [39,40,41].

As shown in Table 1, the SDIF histogram mainly includes TOA difference histogram analysis and impulse sequence searching. The TOA difference histogram analysis is used to estimate PRI values. The histogram statistics method is used to calculate the number of TOA differences between one level and a higher level. If the number of TOA differences exceeds the detection threshold, the TOA difference corresponding to the peak divided by the statistical series of TOA differences is the possible PRI value. The threshold function of the SDIF algorithm can be expressed as:

(18) $$T_{thre}\left(\tau \right)=a\left(E-C\right)e^{-\tau /kN}$$

where *E* is the total number of pulses; *C* denotes the order of the histogram; *k* is a positive constant less than 1; and *N* is the total number of bins in the histogram. The optimum values of the constants *a* and *k* are experimentally determined.

In the actual signal sorting process of the SDIF sorting algorithm, the influence of the intercept receiver on pulse TOA measurement should be avoided to improve the sorting performance of the algorithm for jitter signals. Therefore, the tolerance ε of the PRI value τ is set in the SDIF algorithm and its improved sorting algorithm. The interval of the signal PRI value τ is determined by the tolerance; i.e., the PRI interval or PRI small box. The upper and lower limits of the small box can be expressed as:

(19) $$\tau_{\max}=\tau +\varepsilon$$

(20) $$\tau_{\min}=\tau -\varepsilon$$

Then, the box range of the PRI value is $\tau_{\min}\leq \tau \leq \tau_{\max}$. The PRI value of the histogram is the weighted average of the values that fall in the PRI box. Hence, the weighted average function should be:

(21) $$\bar{\tau }=\sum_{i=1}^{n}\left(x_{i}/S\right)\cdot \tau_{i}$$

where *S* is the sum of the pulse number corresponding to adjacent PRI values $\tau_{1},\tau_{2},\cdot \cdot \cdot ,\tau_{i}$ within the tolerance, and $x_{i}$ is the number of the PRI value $\tau_{i}$ corresponding to pulses within tolerance.

#### 3.2.2. Sorting Failure Principle

##### Analysis of the Sorting Failure Principle of the First-Order Histogram

When the signal is a fixed-period signal—i.e., $\tau =\tau_{0}$—the number of pulses *E* is assumed to be $E_{0}$; $e^{-\frac{\tau }{kN}}$ and *a* are positive constants that are less than 1 when τ>0 in the threshold equation (Equation (18)). The number of pulses is $E_{0}\gg 1$ in the actual situation. Therefore, the right part of the threshold equation is $a\left(E_{0}-C\right)e^{-\frac{\tau }{kN}}<E_{0}$, and the threshold equation is $T_{thre}\left(\tau_{0}\right)<E_{0}$ at $\tau =\tau_{0}$. The signal PRI value $\tau =\tau_{0}$ can be sorted out.

① The number of PRI values increases from 1 to a finite number

Assuming that the total number of pulses $E=E_{0}$ is constant, the signals are two staggered signals when the number of PRI values increases from 1 to 2. In addition, the PRI value is $\tau_{0}$ or $\tau_{1}$ ($\tau_{0}<\tau_{1}$). There are $\frac{E_{0}}{2}$ pulses in each PRI value. The relationship between the number of corresponding pulses and the threshold value $T_{thre}\left(\tau_{0}\right)$ when the PRI value and pulse number change is discussed next.

According to Equation (18), when the PRI value is $\tau =\tau_{0}$, the threshold of the algorithm should be:

(22) $$T_{thre}\left(\tau_{0}\right)=a\left(E_{0}-1\right)e^{-\tau_{0}/kN}$$

When the PRI value is $\tau =\tau_{1}$, the threshold of the algorithm can be expressed as:

(23) $$T_{thre}\left(\tau_{1}\right)=a\left(E_{0}-1\right)e^{-\tau_{1}/kN}$$

When the threshold of the algorithm is $\frac{E_{0}}{2}$—i.e., $T_{thre}\left(\tau^{\prime }\right)=\frac{E_{0}}{2}$—the PRI critical value of the sorting failure can be calculated with Equation (24):

(24) $$a\left(E_{0}-1\right)e^{-\tau^{\prime }/kN}=\frac{E_{0}}{2}$$

where $E_{0}$ represents the total number of pulses (usually in the tens of thousands); i.e., $E_{0}\gg 1$. Therefore, Equation (24) can be simplified as:

(25) $$aE_{0}e^{-\tau^{\prime }/kN}=\frac{E_{0}}{2}$$

Therefore, after taking the natural logarithm of the above equation, $\tau^{\prime }$ can be obtained:

(26) $$\tau^{\prime }=kN\ln\left(2a\right)$$

It can be known from the monotonically decreasing property of the threshold function that the SDIF algorithm can sort out the pulses of PRI $\tau_{0}$ when $\tau_{0}\geq kN\ln\left(2a\right)$; i.e., $\frac{E_{0}}{2}\geq T_{thre}\left(\tau_{0}\right)$. The SDIF algorithm can also sort out the pulses of PRI due to $\tau_{0}<\tau_{1}$; the SDIF algorithm cannot sort out the pulses of PRI $\tau_{0}$ when $\frac{E_{0}}{2}<T_{thre}\left(\tau_{0}\right)$. However, if $\tau_{1}\geq kN\ln\left(2a\right)$, the SDIF algorithm can sort out the pulses of PRI $\tau_{1}$, $\frac{E_{0}}{2}\geq T_{thre}\left(\tau_{1}\right)$; if $\tau_{1}<kN\ln\left(2a\right)$, the SDIF algorithm cannot sort out them. Consequently, the SDIF algorithm cannot sort the signal pulse with a PRI less than the critical value kNln(2a) when the number of PRI values increases from 1 to 2.

According to the above analysis, the actual signal sorting situation can be extended. The derivation condition is assumed as follows:

(1) The total number of pulses is still $E_{0}$;
(2) The number of PRI values increases finitely, meaning that the signals are multiple staggered signals;
(3) The PRI values are $\tau_{1}$, $\tau_{2}\cdot \cdot \cdot \tau_{n}$($\tau_{1}<\tau_{2}<\cdot \cdot \cdot <\tau_{n}$);
(4) The number of pulses corresponding to each PRI value is $\frac{E_{0}}{n}$.

The derivation process is the same as that of Equations (22)–(26) and will not be repeated here. The PRI critical value for the sorting failure can be solved as follows:

(27) $$\tau^{\prime }=\mathrm{kN}\ln\left(na\right)$$

Finally, the SDIF algorithm cannot select a signal pulse with a PRI less than the critical value kNln(na) when the number of PRI signals increases to a finite number.

② The signal PRI value follows an interval distribution

The number of pulses *E* is assumed to be $E_{0}$. The signal PRI value follows an interval distribution $\tau \in \left[\tau_{0},\tau_{1}\right]$. The design of transmitting signals and the processing of echo signals need to be considered in the radar field. The PRI value of the radar signal does not follow a completely random, disordered distribution. Hence, this section discusses the PRI values of signals with a uniform distribution. For any given interval, the number of pulses $E^{\prime }$ is:

(28) $$E^{\prime }=\frac{E_{0}}{\left(\tau_{1}-\tau_{0}\right)/z}$$

where $E_{0}$ is the total number of pulses; $\tau_{1}$ and $\tau_{0}$ denote the maximum and minimum values of the distribution interval; and z is the minimum interval of PRI values within the interval distribution. The threshold calculation equation for the algorithm when PRI is $\tau^{\prime }$ is shown in Equation (18).

When $T_{thre}\left(\tau^{\prime }\right)=E^{\prime }$,

(29) $$a\left(E_{0}-1\right)e^{-\tau^{\prime }/kN}=\frac{E_{0}}{\left(\tau_{1}-\tau_{0}\right)/z}$$

where $E_{0}$ is the total number of pulses, $E_{0}\gg 1$. Therefore, the above equation can be simplified as:

(30) $$aE_{0}e^{-\tau^{\prime }/kN}=\frac{E_{0}}{\left(\tau_{1}-\tau_{0}\right)/z}$$

Then, the PRI critical value for sorting failure can be obtained as follows:

(31) $$\tau^{\prime }=kN\ln\left(\frac{a\cdot \left(\tau_{1}-\tau_{0}\right)}{z}\right)$$

When $\tau_{1}<\tau^{\prime }$, the number of pulses corresponding to PRI values within the interval $\left[\tau_{0},\tau_{1}\right]$ is less than the threshold value. Hence, the SDIF sorting fails. When $\tau_{0}<\tau^{\prime }<\tau_{1}$, the number of pulses corresponding to the PRI value within the interval $\left[\tau_{0},\tau^{\prime }\right)$ is less than the threshold value. Therefore, the SDIF sorting also fails within the interval $\left[\tau_{0},\tau^{\prime }\right)$. When the number of pulses corresponding to the PRI value within the interval $\left[\tau^{\prime },\tau_{1}\right)$ is greater than the threshold value, the signal can be sorted successfully. When the PRI value of the signal follows the interval distribution, the SDIF algorithm cannot select the signal pulse with a PRI less than the critical value $kN\ln\left(\frac{a\left(\tau_{1}-\tau_{0}\right)}{z}\right)$.

The conditions for the actual signal sorting process can be described as follows:

(1) *N* is the number of cells counted in the histogram and is usually above 1000;
(2) *z* is the minimum interval of the PRI. In general, w, $\tau_{1}$, and $\tau_{0}$ have the same size scale at the microsecond level;
(3) a, k ∈(0,1) in Equation (18).

Therefore, when the interval length is 20 times larger than *z*, we know that $\ln\left(\frac{a\cdot \left(\tau_{1}-\tau_{0}\right)}{z}\right)>1$. The threshold value is much larger than any value in the interval $\left[\tau_{0},\tau_{1}\right]$, i.e., $\tau^{\prime }\gg \tau_{1}$.

In conclusion, when the signal PRI values follow the interval distribution and the interval length is 20 times larger than *z*, the number of pulses corresponding to any PRI value in the interval $\left[\tau_{0},\tau_{1}\right]$ is less than the sorting algorithm threshold in the analysis of the sorting failure principle of the first-order histogram. Hence, the SDIF algorithm fails in signal sorting.

##### Analysis of the Sorting Failure Principle in a Multi-Order Histogram

The SDIF sorting algorithm sometimes has multiple PRI values exceeding the threshold in the first-order histogram. Thus, it is necessary to count the second-order, third-order, and advanced histograms to produce PRI estimates. Moreover, the sorting failure principle for second-order to advanced histograms also needs to be analyzed.

The threshold function is shown in Equation (18). From second-order to multi-order histograms, only the histogram series *C* changes. The total number of pulses *E* is much larger than *C*; i.e., E≫C.

Thus, the threshold function is approximately unchanged. When the signal PRI value follows the interval distribution, the first-order histogram in Section 3.2.2 is still applicable for the analysis of the sorting failure principle of the SDIF algorithm.

In summary, when the radar signal PRI values obey a interval distribution with a length greater than 20 times the minimum interval of the PRI, the accumulation of the signal pulse difference histograms at all levels is lower than the sorting algorithm threshold, leading to a sorting algorithm failure.

### 3.3. Signal Design Principles

The sorting failure principles for the pre-sorting clustering algorithm and the main SDIF sorting algorithm were analyzed in Section 3.1 and Section 3.2, respectively. The failure principles for the two sorting algorithms were obtained. The failure principle for the clustering sorting indicates that, when the parameter center value of any dimension of two adjacent intervals is at least half of the sum of the intervals in each dimension, relatively independent multiple pseudo-cluster centers without overlap can be formed. The SDIF sorting failure means that, when the radar signal PRI value follows an interval distribution with a length more than 20 times the minimum interval of the PRI, the accumulation of signal pulse difference histograms at all levels is lower than the threshold of the sorting algorithm, and the SDIF sorting algorithm fails. The clustering sorting failure actually “fakes” the wrong correlation between signal pulses through wide-interval agility and “cheats” the clustering sorting algorithm. In contrast, the SDIF sorting failure reduces the correlation between signals by submitting the PRI values of the radar signals to an interval distribution with a length more than 20 times greater than the minimum PRI interval. As a result, the cumulation of signal pulse difference histograms at all levels is lower than the threshold of the sorting algorithm.

To sum up, the essence of signal sorting is “matching” the pulse sequences belonging to the same radiation source through the correlation between pulses in the pulse flow to achieve signal sorting. The essence of RF stealth signal design consists of reducing the correlation between pulse sequences and strengthening the randomness between pulses. Since a signal sorting algorithm is designed for thousands of nearly disordered random pulse streams, not all low-correlation random pulse sequences have anti-sorting abilities, thus requiring a targeted design based on clustering and SDIF sorting failure principles. A chaotic sequence is characterized by strong and good randomness. Therefore, this paper proposes a design method for anti-sorting signals based on a 2D hyperchaotic system using chaotic sequences according to the principles of clustering and SDIF sorting failure.

The method for anti-sorting signal design proposed in this paper mainly aims at the main SDIF sorting algorithm and the clustering pre-sorting method based on data fields. Despite that, from the analysis of the signal design principle, it can be deduced that the stronger the randomness of the anti-sorting signal, the better its anti-sorting performance. Therefore, the anti-sorting signal designed here can improve not only the anti-sorting performance of the SDIF and clustering algorithms based on data fields mentioned in this paper but also the resistance abilities of other sorting algorithms.

## 4. Anti-Sorting Signal Design Based on a 2D Hyperchaotic System

### 4.1. Construction of 2D Hyperchaotic Systems

Based on the classical Henon 2D chaotic system, the cosine function and exponential function were introduced into the mapping functions of the X and Y dimensions, respectively, to improve the design, as shown in Equation (32):

(32) $$\begin{array}{l} x_{n+1}=y_{n}+1-ax_{n}^{2}(a\in (0,1.4))\\ y_{n+1}=bx_{n}(b\in \left(0.2,0.314\right]) \end{array}$$

The main elements of chaotic mapping are the cosine and sine functions of the trigonometric function. The mapping design of the trigonometric function is divided into X and Y dimensions. In the X dimension, there are $\pi rx_{n}e^{x_{n}}\left(1+x\right)$ and $\pi \left(e^{x_{n}}+x^{3}\right)$ in the cosine function. While $\pi rx_{n}e^{x_{n}}\left(1+x\right)$ is the main element that produces the chaotic sequence, $\pi \left(e^{x_{n}}+x^{3}\right)$ further enhances the randomness and unpredictability of the chaotic sequence generated in $\pi rx_{n}e^{x_{n}}\left(1+x\right)$ through exponential and higher-order terms. In the Y dimension, the sine function consists of $\pi ry_{n}\left(1-\tan y_{n}\right)$ and $\pi e^{x_{n}}$. While $\pi ry_{n}\left(1-\tan y_{n}\right)$ is the main element that produces the chaotic sequence, $\pi e^{x_{n}}$ enhances the randomness and unpredictability of chaotic sequences. In addition, to endow the chaotic system with more complex chaotic dynamics characteristics, the outputs of the X and Y dimensions are fed back to the other dimension in an interleaving feedback manner to make them participate in the iterative calculation of the X and Y dimensions at the next moment. The chaotic mapping is expressed in Equation (33):

(33) $$\begin{array}{l} x_{n+1}=\cos\left[\pi ax_{n}e^{x_{n}}\left(1+x_{n}\right)+\pi \left(e^{y_{n}}+y_{n}^{3}\right)\right]\\ y_{n+1}=\sin\left[\pi ry_{n}\left(1-\tan y_{n}\right)+\pi e^{x_{n}}\right] \end{array}$$

**Figure 2:**
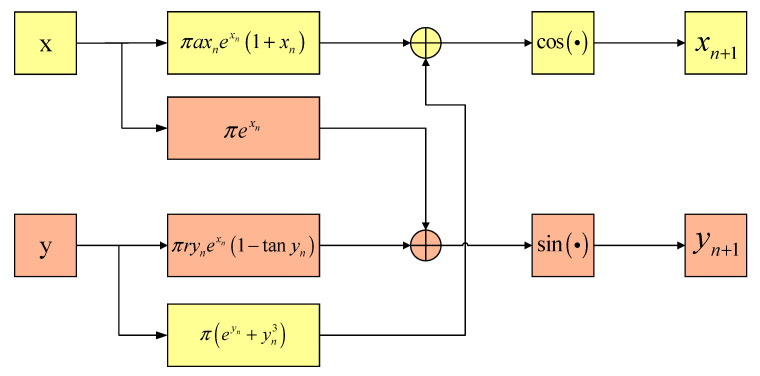
Block diagram of the proposed chaotic mapping.

Based on classical Henon mapping, sine and cosine functions can be introduced to control the output of the chaotic mapping within [−1,1]. As a result, the parameter center value of the signal can correspond to the output center value of the chaotic mapping 0, and the mapping output values 1 and −1 can correspond to any range limit of the parameter variation. The exponential items $\pi e^{x_{n}}$ and $\pi e^{y_{n}}$ in the second part eliminate the restriction that the initial value cannot be 0, making the generation of chaotic sequences more flexible. Moreover, the entry of the system into a chaotic state is no longer limited by the control coefficient. Thus, a system can enter a chaotic state more quickly. In the Y dimension, the cubic term $\pi y_{n}^{3}$ is not used to further increase the randomness, as the high randomness of the X dimension can be transmitted to the Y dimension through the interleaving feedback of the two dimensions. Synchronous, high randomness in the X and Y dimensions can be achieved with fewer computing resources and less time. A block diagram of the chaotic mapping proposed in this paper is shown in Figure 2.

**Figure 3:**
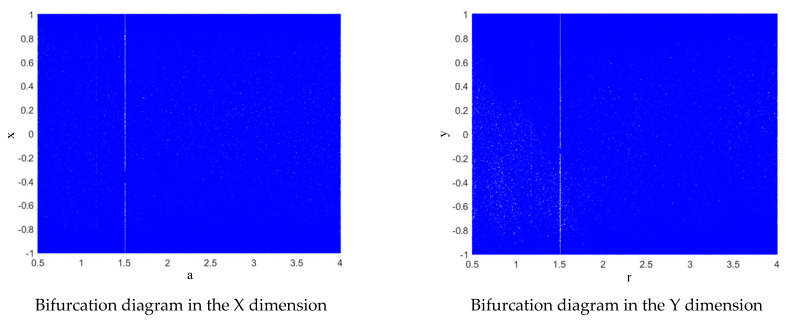
Bifurcation diagrams of the proposed chaotic mapping.

The block diagram of the chaotic mapping intuitively reflects the interleaving feedback process of the X and Y dimension sequences of the chaotic mapping proposed in this paper. The interleaving feedback helps to enhance the randomness of the two dimensions. The chaotic mapping bifurcation diagrams proposed in this paper are shown in Figure 3.

### 4.2. Design Method for Wide-Interval Signals

In anti-clustering signal design, it is necessary to use a joint design method for the 3D pseudo-center width agility based on the interval distribution. The interval length of the PRI variation is 20 times the minimum interval length in the anti-PRI signal design in Section 3.3. Therefore, the PW, RF, DOA, and PRI all need to achieve agility with the two separation failure principles. In contrast, DOA agility is mainly achieved with antenna design technology, independently of the signal parameter design. If the stop time of the pulse signal is kept constant, the change in the PRI causes a change in the PW. Hence, when the PRI and RF are changed, the RF, PW, PRI, and DOA of the designed signal are changed.

The X dimension of the chaotic mapping is used to modulate the PRI value *t*, and the RF value *f* of the signal is modulated by the Y dimension. Since the modulation methods are the same, the modulation of the PRI value *t* by the X dimension of the chaotic mapping is taken as an example in the following. The modulation function is shown in Equation (34):

(34) $$t_{n}=t_{0}+x_{n}\cdot \Delta t\left(n\in \left[1,w\right]\right)$$

In Equation (34), $t_{0}$ is the central value of the signal PRI; Δt denotes the maximum variation of the signal PRI; $x_{n}$ is generated by the chaotic mapping of Equation (33); *w* indicates the maximum modulation times of the PRI in an interval. After *w* modulations in any change interval, the PRI center value becomes agile and will be modulated in another interval to realize the pseudo-clustering center and “trick” the clustering algorithm.

**Figure 4:**
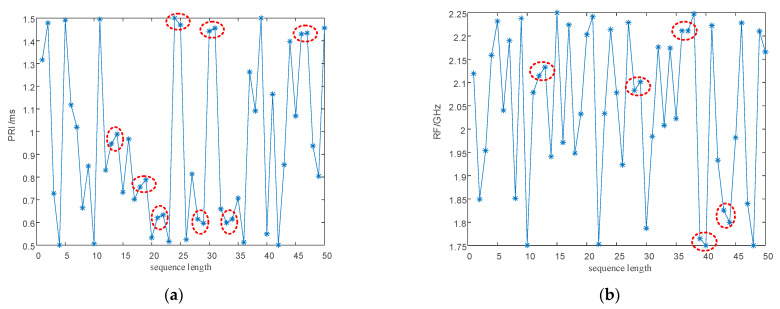
The narrow, multi-value interval effect. (**a**) The narrow, multi-value interval effect of the PRI sequence. (**b**) The narrow, multi-value interval effect of the RF sequence.

After modulation, PRI and RF value sequences retain the randomness of chaotic sequences. However, as shown by the red circle in Figure 4, within a change interval, the PRI and RF value sequences modulated by the chaotic mapping have narrow, multi-value interval effects; that is to say, there are several adjacent PRI and RF values in the sequence with small intervals, resulting in adjacent pulses falling into the same pulse-sorting tolerance.

Therefore, before chaotic sequences modulate the PRI and RF values, the method of generation for chaotic sequences needs to be improved to eliminate the narrow, multi-value interval effect. During the generation of a sequence from a chaotic mapping, each time a sequence value $x_{n}$ is generated by the chaotic mapping, the interval between $x_{n}$ and the previous value $x_{n-1}$ must be judged first. If the interval meets the requirements, the value $x_{n}$ is retained, $x_{n}$ is substituted into the chaotic mapping expression for the next iteration to generate $x_{n+1}$ and then the interval between $x_{n+1}$ and $x_{n}$ is judged and so on. If the interval does not meet the requirements, $x_{n}$ is discarded in order to judge whether the interval between $x_{n-1}$ and $x_{n+1}$ generated by an iteration of $x_{n}$ meets the requirements. If the interval requirements are met, $x_{n+1}$ is retained; if not, $x_{n+1}$ is abandoned, and $x_{n-1}$, and $x_{n+2}$ are compared until the chaotic sequence values meet the requirements. In addition, to filter out some of the points concentrated at the boundary of the sequence values, the critical value judgment steps $x_{n}+\Delta x<1$ and $x_{n}-\Delta x>-1$ are added to the sequence point values that meet the interval requirements. When the chaotic mapping modulates *w* times in the PRI interval, the PRI interval becomes agile, and the chaotic mapping continues to modulate *w* times in the next PRI interval and so on.

**Figure 5:**
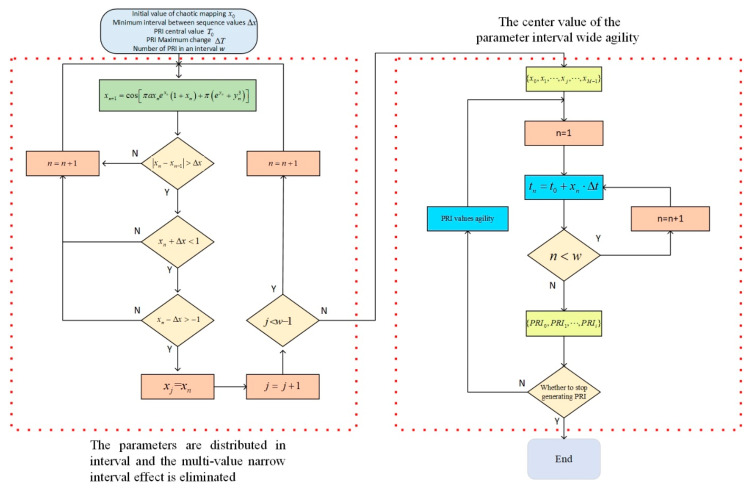
Multi-parameter, composite, wide-interval modulation diagram.

To sum up, the wide and rapid change of the PRI interval to another PRI interval after *w* modulation is intended to obtain the pseudo-clustering center and achieve anti-clustering sorting. Within each defined PRI interval, the PRI follows the interval distribution and eliminates the narrow, multi-value interval effect of the PRI to increase the randomness between signal pulses and achieve anti-SDIF sorting. Taking signal PRI generation as an example, the specific generation process for signal parameters is shown in Figure 5.

**Figure 6:**
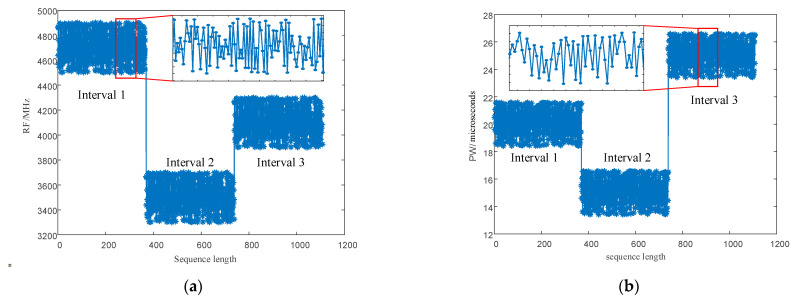
Multi-parameter, composite, wide-interval modulation sequence point diagram. (**a**) RF sequence point plot. (**b**) PRI sequence point plot.

Point diagrams of the PRI and RF numerical sequences generated according to the signal parameter generation process designed in this study are shown in Figure 6. As shown in the figure, wide agility is realized between the three intervals to achieve anti-clustering sorting. As shown in the enlarged local part, the narrow, multi-value interval effect is eliminated in each interval, and the probability of signal sorting by the SDIF sorting algorithm is reduced.

## 5. Simulation and Analysis

The simulation environment used in this study was a Windows 10 64-bit operating system. The computer parameters used as the simulation platform were as follows: the processor was a core I7-9750H, the main frequency was 2.60 GHz, and the memory was 8 GB.

### 5.1. Simulation of the Signal Design Principle

The principle for the signal design provides theoretical support for the design of anti-sorting signals. Therefore, the correctness of the signal design principle needs to be verified with simulations.

#### 5.1.1. Simulation of the Anti-Clustering Signal Design Principle

According to the principle of the anti-clustering signal design, for n-dimensional parameters, when the magnitude of the parameter center dexterity of any dimension of two adjacent intervals is at least half of the sum of the intervals in their respective dimensions, relatively independent multiple pseudo-clustering centers without overlap can be formed. In particular, if the interval values of the parameter distributions of any dimension of two adjacent intervals are equal, relatively independent multiple pseudo-cluster centers without overlap can be formed when the parameter center value of any defined dimension of the adjacent intervals is at least one interval. To verify the correctness and significance of the signal design principle for the signal design, a comparative simulation was conducted. The simulation parameters were set as shown in Table 2.

**Figure 7:**
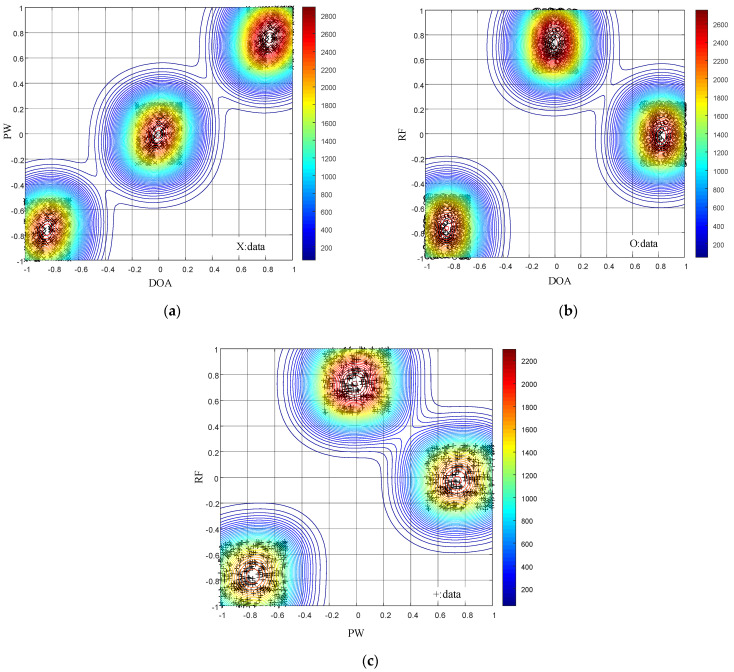
Two-dimesional (2D) distribution diagram of data field isopotential lines for the proposed 3D pseudo-center wide agility joint design. (**a**) The 2D plot for the PW-DOA data field isopotential lines. (**b**) The 2D plot for the RF-DOA data field isopotential lines. (**c**) The 2D plot for the RF-PW data field isopotential lines.

**Figure 8:**
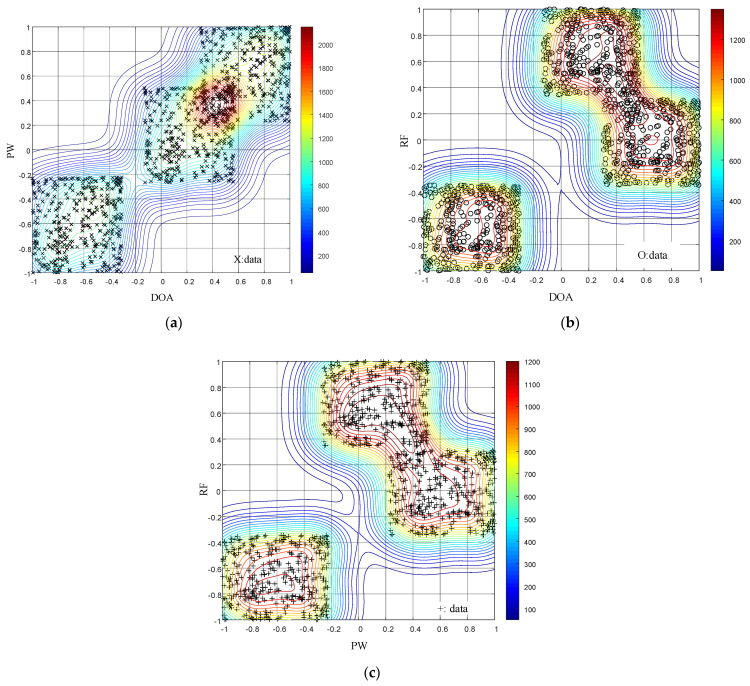
The 2D distribution of data field isopotential lines for the 3D pseudo-center wide agility joint design. (**a**) The 2D plot for the PW-DOA data field isopotential lines. (**b**) The 2D plot for the RF-DOA data field isopotential lines. (**c**) The 2D plot for the RF-PW data field isopotential lines.

The results for the data field clustering algorithm are shown in Figure 7 and Figure 8.

As shown in Figure 7, using the parameter generation method for the 3D pseudo-center wide agility joint design proposed in this paper, the data field clustering algorithm sorts the signals sent by the radar into three clustering centers in the PW-DOA, RF-DOA, and RF-PW dimensions. The clustering algorithm is successfully “tricked”, and algorithm sorting fails. As shown in Figure 8, the anti-clustering algorithm proposed in this paper is not used to design signal parameters. The RF-DOA and RF-PW dimensions are sorted into two clustering centers, and the PW-DOA dimension is sorted into one clustering center. The clustering algorithm can perform correct sorting in the PW-DOA dimension but cannot achieve anti-clustering sorting. By comparing Figure 7 and Figure 8, the correctness of the signal design principle can be verified, proving that the design principle provides strong theoretical support for anti-sorting signal design.

#### 5.1.2. Simulation of the Anti-SDIF Signal Design Principle

According to the design principle for the anti-SDIF signal, when the designed radar signal PRI value follows an interval distribution with a length of more than 20 times the minimum interval PRI, the signal sorting fails. Therefore, when designing signals, the PRI interval length should be at least 20 times greater than the minimum interval. To verify the correctness and significance of the signal design principle, a comparative simulation was conducted, which is described in this section. The simulation parameters were set as follows.

**Figure 9:**
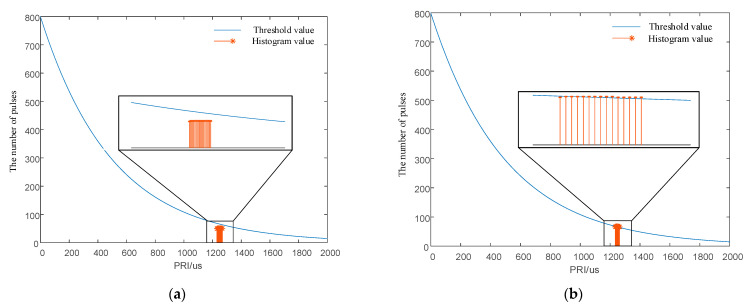
Simulation verification diagrams for the signal design principle. (**a**) First-order TOA difference histogram with a PRI interval length 20 times the minimum interval. (**b**) First-order TOA difference histogram with a PRI interval length 15 times the minimum interval.

The signal was 1000 pulses in total, and the TOA measurement error was 50 μs. The PRI center value was 1250 μs, and the minimum PRI interval was 2 μs. The interval length of the PRI was 20 and 15 times the minimum interval; i.e., the PRI interval was [1230 μs, 1270 μs] and [1235 μs, 1265 μs]. First-order histogram analysis ranged from 0 to 2500 μs, and the statistical interval of the histogram was 0.5 μs. The SDIF statistical threshold is shown in Equation (18), where *N* = 5000, *k* = 0.1, and *a* = 0.8. The SDIF sorting results are shown in Figure 9.

As shown in Figure 9a, when the interval length of the signal PRI is greater than or equal to 20 times the minimum interval, the pulse statistics corresponding to any PRI value within the interval are smaller than the threshold of the SDIF algorithm, and the algorithm fails. Figure 9a also shows that the signal designed in this paper still has sorting resistance, with a small jitter amplitude of only 1.6%. Additionally, when the interval length of the signal PRI is less than 20 times the minimum interval, the numbers of individual signal pulses within the interval exceed the threshold and can be sorted successfully by the SDIF algorithm. The simulation verifies the correctness of the signal design principle and proves that the design principle provides strong theoretical support for anti-sorting signal design.

### 5.2. Performance Simulation of Hyperchaotic Systems

Utilizing the principle of the signal design, an anti-sorting signal was designed with a chaotic system in this study. Signal parameters are modulated by the chaotic system, the performance of which determines the anti-sorting performance of the signals. Therefore, it is necessary to compare the chaotic system proposed in the paper with other mappings.

#### 5.2.1. Chaotic Mapping Performance Analysis

To further illustrate the advantages of the hyperchaotic system designed in this paper, typical 1D logistic mapping, 2D Henon mapping, and 3D Lorenz mapping were selected for comparison with the proposed mapping. The bifurcation diagram, maximum Lyapunov index, and approximate entropy of the chaotic mapping were also compared.

**Figure 10:**
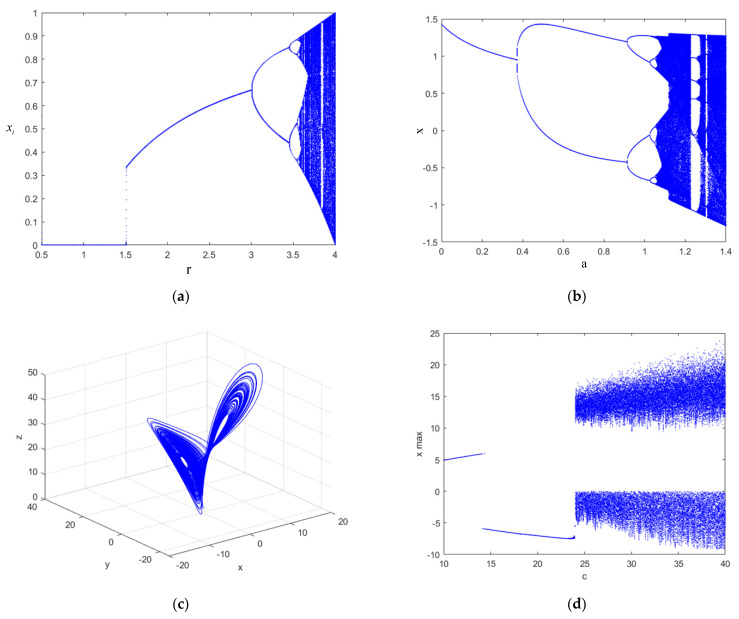
Bifurcation diagrams for chaotic mappings. (**a**) Bifurcation diagram for the logistic mapping. (**b**) Bifurcation diagram for the Henon mapping. (**c**) Phase space diagram for the Lorenz mapping. (**d**) X-dimensional bifurcation diagram for the Lorenz mapping.

The bifurcation phenomenon in chaotic mapping is one of the signs that the mapping has entered a chaotic state. By depicting the bifurcation diagram of the mapping, the chaotic mapping region and the influence of the control parameters in the mapping on the chaos can be observed intuitively. For the Lorenz high-dimensional chaotic system, the phase space diagram can also intuitively display its chaotic state. The bifurcation diagram and phase space diagram were drawn according to the expressions for the three mappings (logistic mapping, Henon mapping and Lorenz mapping), as shown in Figure 10. And the chaotic mapping bifurcation diagrams proposed in this paper are shown in Figure Figure 3.

As shown in the figure, when the fractal coefficient of the logistic mapping is r≥3.57, the system enters a chaotic state. When 0≤r<3.57, the chaotic system is periodic. When the Henon mapping has the fractal coefficient a≥1.1, the system enters a chaotic state. When 0≤r<1.1, the system is in a periodic state. When the X dimension of the Lorenz mapping is at c≥24, the system enters a chaotic state. When 0<c<24, the system is in a periodic state. The chaotic map designed in this paper is not affected by the fractal coefficient, and the points in the bifurcation diagram are more evenly distributed.

**Figure 11:**
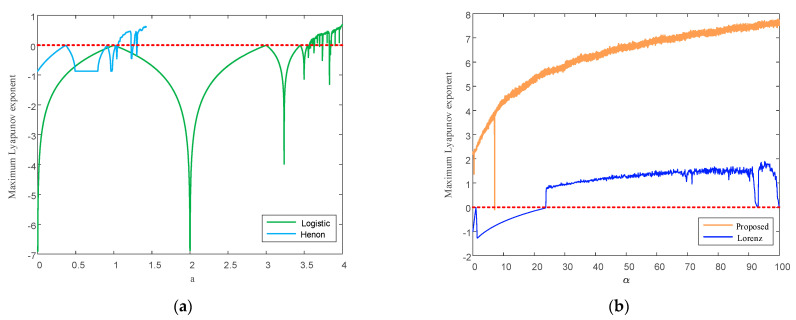
LE comparison diagrams of different chaotic mappings. (**a**) Maximum LEs of logistic and Henon mappings. (**b**) Maximum LEs of the Lorenz mapping and the proposed mapping.

The maximum Lyapunov exponent (LE) was used to evaluate the randomness of chaotic sequences, and its definition is shown in Equation (35):

(35) $$LE=\underset{n\to \infty }{\lim}\left(\frac{1}{n}\sum_{i=0}^{n-1}\ln\left|f^{\prime }\left(x_{i}\right)\right|\right)$$

where $f^{\prime }\left(x_{i}\right)$ represents the first derivative of the chaotic mapping $f\left(x_{i}\right)=x_{n+1}$. When LE>0, the system is in a chaotic state. The larger its value is, the stronger the randomness of the chaotic sequence generated by the system is. The LE curves of the above four chaotic maps with different fractal coefficients are plotted below. The variation range for the fractal coefficient of each chaotic mapping is different. Therefore, to fully demonstrate the variation trend for each chaotic mapping with the fractal coefficient, the LEs of the logistic and Henon chaotic mappings, as well as the LEs of the Lorenz mapping and the proposed mapping, were drawn in the same graph, as shown in Figure 11.

As shown in Figure 11, the LEs of the logistic, Henon, and Lorenz mappings are not always greater than 0, indicating that the three systems are not always in a chaotic state. Taking the logistic mapping as an example, only when r≥3.57 is the system in chaos. In the fractal coefficient variation interval [0,100], the LE of the chaotic mapping proposed in this paper is greater than 0, except for one point less than 0, thus avoiding the limitation of the fractal coefficient. In addition, the LE of the chaotic mapping designed in this paper is greater than that of the other three chaotic mappings, indicating that the chaotic sequence generated by the proposed chaotic mapping has stronger randomness than those generated by the other three classical chaotic mappings.

The approximate entropy measures the complexity of the chaotic sequence generated by the chaotic mapping. The higher the approximate entropy, the more complex the sequence is. The approximate entropy values for the above four mappings were calculated under the same simulation conditions, where the values of the fractal coefficient varied from 0.5 to 4 with a step size of 0.5 and the sequence length was set as 10,000.

It can be seen from the calculation results in Table 3 that when the system does not enter the chaotic state, the approximate entropy value is minimal or even 0. For example, when r=0.5 or r=1, the approximate entropy of the logistic mapping is of the order of $10^{-5}$ or $10^{-7}$. However, the chaotic mapping proposed in this paper maintains an approximate entropy value greater than 1 in the interval of the fractal coefficient variation, indicating that the sequence generated by the mapping has higher complexity.

**Figure 12:**
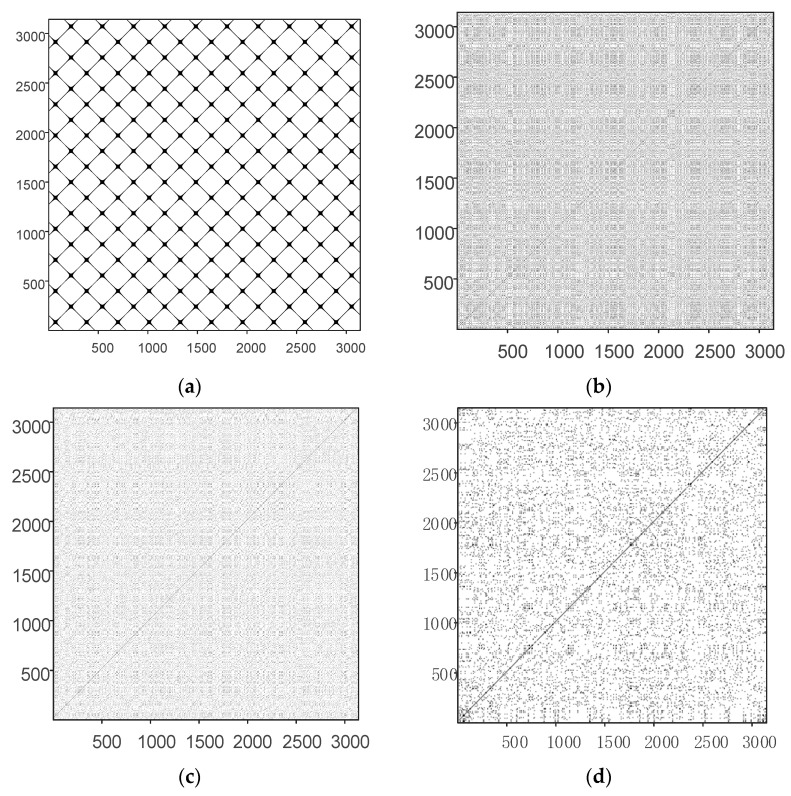

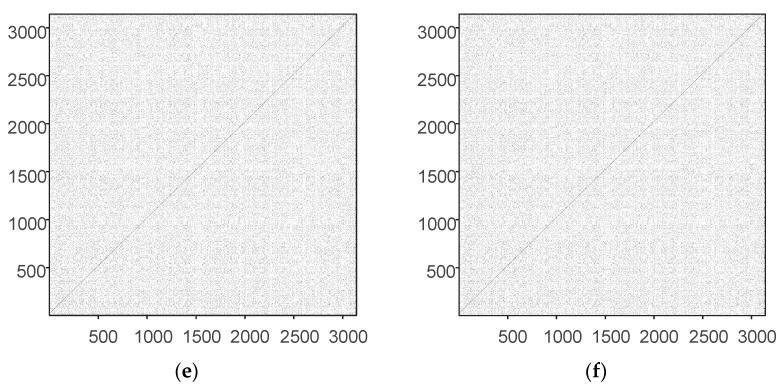
The RPs of different sequences. (**a**) RP of sinusoidal sequence. (**b**) RP of Logistic sequence. (**c**) RP of Henon sequence. (**d**) RP of Lozenz sequence (Y dimension). (**e**) RP of the proposed chaotic mapping sequence (X dimension). (**f**) RP of the proposed chaotic mapping sequence (Y dimension).

The RP is a non-stationary signal processing and analysis method widely used for analysis of chaotic sequences and modulation signals based on chaotic sequences. The RP is drawn from a recursive matrix, as shown in Equation (36):

(36) $$R_{i,j}\left(\varepsilon \right)=\Theta \left(\varepsilon -\left\|{\vec{\eta }}_{i}-{\vec{\eta }}_{j}\right\|\right)\;i,j=1,2,\cdot \cdot \cdot ,N$$

where $R_{i,j}$ is the square matrix of N×N, *N* is the number of the state vector ${\vec{\eta }}_{i}$, the threshold ε represents the pre-set critical distance, ‖·‖ denotes the norm, and Θ(·) is the unit step function, Θ(x<0)=0 and Θ(x>0)=1. The RP is a recursive matrix drawn in different colors in binary. The chaotic signal is analyzed by the RP, and the rule for the chaotic signal is obtained. Periodic or quasi-periodic recurrent structures, such as checkerboard structures, appearing the diagonal direction of the RP characterize the period of the signal state evolution. The randomness of the signal is good if all the recurrence points in the RP are isolated and follow a uniform distribution with no more diagonal, vertical, or horizontal lines. The RP of the sinusoidal sin(2πt) sequence; the sequences generated by the logistic, Henon, and Lorenz mappings; and the sequence generated by the chaotic mapping designed in this paper are drawn in Figure 12. There are 3100 point values in each sequence.

The RP of the sinusoidal sequence in Figure 12a presents an obvious grid-like recursion structure, indicating that the sinusoidal sequence has significant periodicity. The RPs of the (b) logistic, (c) Henon, and (d) Lozenz sequences have no significant recurrence structures overall. However, the recurrence points are unevenly distributed, with small, short lines, indicating a short period in the sequence. Figure 12e,f show the RPs of the X and Y mapping dimensions designed in this paper, respectively. There is only one main 45° diagonal, and the rest of the recursion points are scattered over the plot and are perfectly uniform. The density distribution of each local recurrence point is basically the same, which suggests that the chaotic sequence designed in this paper has strong randomness.

RPs are mainly used for qualitative analysis of chaotic sequences. Thus, quantitative analysis and comparison of chaotic sequences using RQA are required. Following the literature [31], the recursive rate (RR), entropy (ENTR), system determinacy (DET), and maximum diagonal length ($L_{\max}$) were used to analyze chaotic sequences. The larger the recurrence rate, the more points are clustered in the RP. The smaller the recurrence rate, the more uniform the distribution of points in the RP of the system is. Larger entropy indicates that the system is more complex, and vice versa. The larger the DET value, the stronger the determinacy of the chaotic sequence is. A smaller DET value indicates stronger randomness. The faster the attractor trajectory diverges in the phase space, the shorter the diagonal is; i.e., the shorter the maximum diagonal.

According to Table 4, the chaotic mapping designed in this paper has the smallest recurrence rate. This result indicates that the points of the mapping designed in this paper are evenly distributed in the RP without aggregation phenomena, which is corroborated by the RP in Figure 12. In terms of system certainty, the DET value of the chaotic mapping designed in this paper is the smallest, close to that of Gaussian white noise. Therefore, the chaotic mapping designed in this paper has strong randomness. Regarding the maximum diagonal length, the diagonal length of the proposed chaotic mapping is very short. In particular, the diagonal length of the Y dimension is the same as that of white Gaussian noise. Therefore, the attractor of the designed chaotic mapping diverges rapidly in the phase space. Finally, the chaotic mapping designed has the largest entropy, much larger than the logistic, Henon, and Lorenz mappings, which again indicates that the proposed chaotic mapping is relatively complex.

Sixth, to test the pseudo-randomness of the X- and Y-dimensional chaotic sequences generated by the designed chaotic mapping, we adopted the NIST-800-22 test scheme. The test scheme contains 15 statistical test items, each of which calculates a *p*-value, which was compared with a given significance level to determine the randomness of the generated sequence. In this experiment, we set the significance level α=0.01 and the test sequence length to 1,000,000. When all test items meet p-value>α, the randomness of the generated sequence is considered to meet the requirements. After several tests, all the test targets were achieved. Table 5 shows the test results for a certain time. It can be seen from the table that the test results for all the test items met the requirements.

Finally, the computational complexity of the chaotic mapping was studied. The parameters of the signal were modulated with the chaotic mapping designed in this paper. The computational complexity of the chaotic mapping determines the speed of the modulation of the signal parameters by the chaotic mapping, thus affecting the modulation mode. If the chaotic mapping takes a short time to generate a chaotic sequence, the signal parameters can be modulated with online real-time modulation. If the chaotic mapping takes a long time to generate chaotic sequences, the signal parameters need to be modulated with offline non-real-time modulation. Therefore, it is necessary to investigate the computational complexity of the chaotic mapping. To directly reflect the computational complexity of the chaotic mapping, the time required to generate 1,000,000 sequence values by the logistic, Henon, and the designed chaotic mappings were compared in the same simulation verification platform, as shown in Table 6.

As shown in Table 5, although the time take by the chaotic mapping designed in this paper was the longest, the difference from the times taken in chaotic sequence generation by the classical logistic and Henon mappings was not very significant (only 0.01088 s and 0.04201 s). However, the designed chaotic mapping far exceeds the classical logistic and Henon mappings in complexity, randomness, and other aspects. Therefore, the chaotic mapping designed in this paper is still meaningful.

#### 5.2.2. Performance Analysis of PRI and RF Sequences

**Figure 13:**
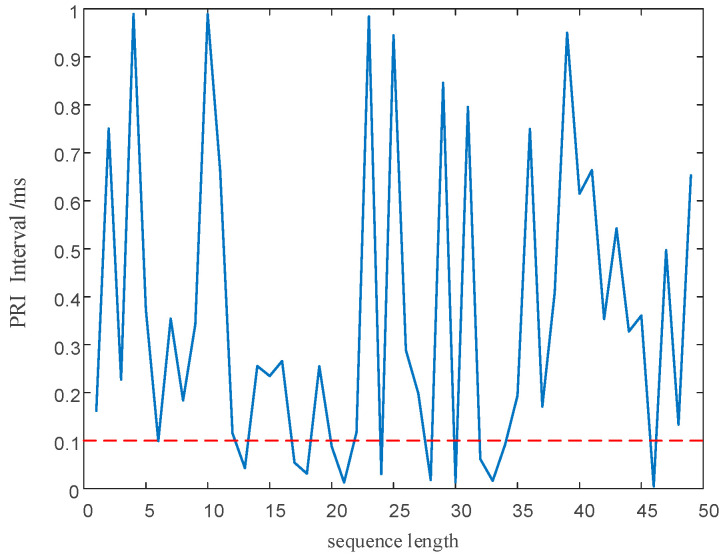
The interval between adjacent PRIs before optimization.

**Figure 14:**
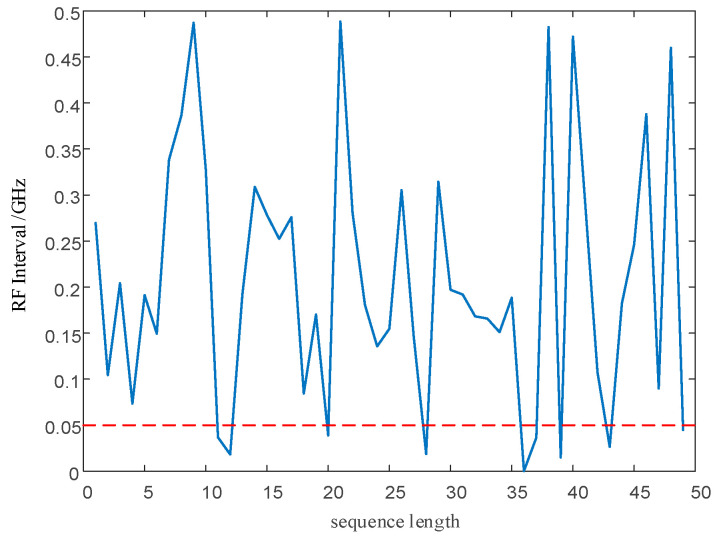
The interval between adjacent PRIs before optimization.

As shown in Figure 13a,b and Figure 14a,b, direct modulation of the signal PRI and RF by the proposed chaotic mapping results in a narrow, multi-value interval effect, which is not conducive to the jump in the PRI and RF values and easily causes the adjacent PRI values to fall into the same PRI tolerance range. Since the modulation processes of the PRI and RF are the same, a chaotic mapping wide-interval modulation method for the PRI and RF can be proposed. The simulation parameters were set as shown in Table 7. The sequence of the PRI and RF before optimization are shown in Figure 4. The interval between adjacent PRIs and RFs before optimization are shown in Figure 13 and Figure 14 respectively.

**Figure 15:**
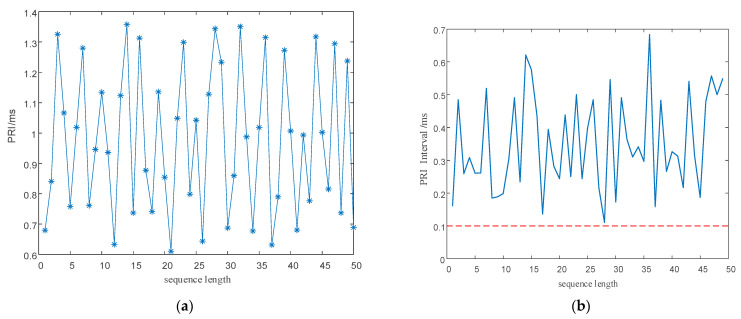
PRI sequence and interval between adjacent PRIs after optimization. (**a**) Optimized PRI sequence. (**b**) Interval between adjacent PRIs after optimization.

**Figure 16:**
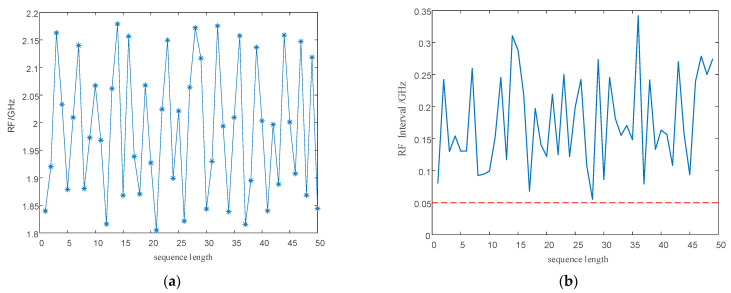
RF sequence and interval between adjacent RFs after optimization. (**a**) Optimized RF sequence. (**b**) The interval between adjacent RFs after optimization.

To eliminate the influence of the initial value of the chaotic system, the chaotic sequence was iterated 1000 times and modulated from the 1001th time. To facilitate simulation analysis, the length of the chaotic sequence was set to 50. The simulation results are shown in Figure 15 and Figure 16.

According to Figure 14 and Figure 16, our proposed method optimizes the generation method for PRI and RF sequences, eliminates the narrow, multi-value interval effect, and satisfies the requirements for PRI and RF difference intervals to enhance the anti-sorting performance of signals. An analysis of the simulation results in Figure 13, Figure 14, Figure 15 and Figure 16 shows the flexibility of the designed signal. First, modulating the PRI and RF of the signals with chaotic sequences can make full use of the initial value sensitivity and fractal coefficient sensitivity of the chaotic system to provide a wider range of PRI and RF values for the signals. Second, the design of the PRI and RF has great flexibility for different working conditions. The PRI and RF sequence can be generated once according to the total length of the designed signal or can be cut into several segments to generate them multiple times, which produces different PRI and RF sequences.

### 5.3. Simulation of Anti-Sorting Performance

This section presents the simulation of the anti-sorting performance for the designed signal, which is mainly divided into anti-clustering sorting and the main anti-SDIF sorting. Since the performance of anti-clustering sorting was simulated and described in Section 5.1.1, the anti-clustering sorting performance of the designed signal was not verified again in this part of the study. The anti-SDIF sorting performance of the signal was verified.

#### 5.3.1. Signal Sorting Simulation Based on the SDIF Algorithm

**Figure 17:**
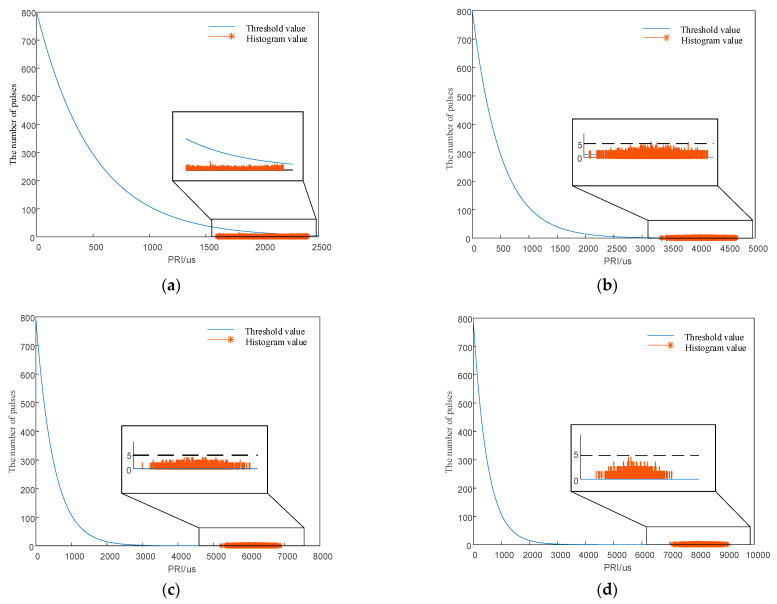
First- to fourth-order TOA difference histograms. (**a**) First-order TOA difference histogram. (**b**) Second-order TOA difference histogram. (**c**) Third-order TOA difference histogram. (**d**) Fourth-order TOA difference histogram.

The simulation parameters were set as follows. The signal amounted to 1000 pulses. The TOA measurement error was 50 nanoseconds. The PRI center value of the signal was 2 ms, with a PRI in the range from 1500 μs to 2500 μs. First-order histogram analysis ranged from 0 to 2500 μs, and the statistical histogram interval was 0.5 μs. The SDIF threshold is expressed in Equation (18), where *N* = 5000, *k* = 0.1, and *a* = 0.8. The SDIF sorting results are shown in Figure 17.

As shown in Figure 17a, no signal exceeded the threshold in the first-order SDIF histogram sorting algorithm, which thus achieved an anti-sorting effect. Figure 17b–d show that, although signal pulses exceeded the threshold in the second-, third-, and fourth-order SDIF histogram sorting algorithms, the cumulative number did not exceed 5. Therefore, the signal pulses did not enter the sorting process. Figure 17a–d show that the signal modulated by the proposed chaotic mapping could an achieve anti-sorting effect.

#### 5.3.2. Comparison with PRI Jitter Signal Sorting Simulation

**Figure 18:**
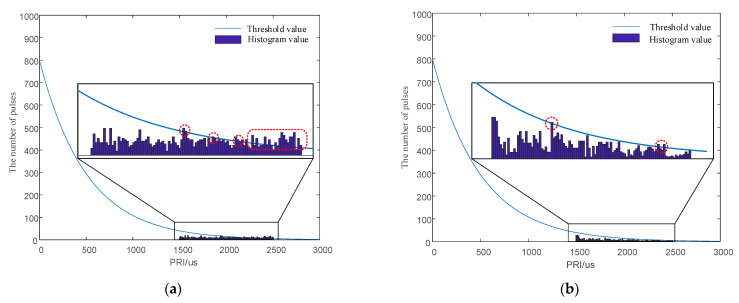
Comparison of diagrams for anti-sorting performance simulation. (**a**) First-order TOA difference histogram for PRI random jitter signals. (**b**) First-order TOA difference histogram for the proposed signals.

For anti-sorting signals, the PRI random jitter signal is also considered a signals with excellent sorting resistance [18]. In this part of the study, the PRI random jitter signal was taken as an example to compare its anti-sorting capability with the signal designed in this paper. The PRI center value of the jitter signal was 2 ms, and its PRI interval was [1500 μs, 2500 μs]. The PRI random jitter signal was the PRI subject to random distribution within [1500 μs, 2500μs]. The designed signal was the PRI generated by the chaotic sequence modulation within [1500 μs, 2500 μs]. The signals to be sorted had 1000 pulses in total. The simulation results are shown in Figure 18.

The histogram for the PRI random jitter signals in Figure 18a exceeded the detection threshold of the sorting algorithm in the area marked by the red circle, and signals were easily sorted by the SDIF algorithm. In Figure 18b, the histogram of the signal TOA difference also exceeded the threshold of the sorting algorithm. However, by comparing their sorting results, it can be seen that the signal modulated by the chaotic sequence under the guidance of the signal design principle analyzed in this paper demonstrated only a few signal values exceeding the threshold in the histogram. However, the histogram of the PRI random jitter signal appeared to exceed the threshold in many cases, indicating that the designed signal is better than the PRI random jitter signal in anti-sorting performance.

## 6. Conclusions

In this study, to enhance the anti-sorting ability of radars, the failure principles of clustering sorting and SDIF sorting were analyzed, and a corresponding signal design method was proposed. Then, the PRI and RF were modulated by the designed hyperchaotic system. Since the pause time of the pulse signals was fixed, the modulation of the PRI, PW, and RF parameters was realized indirectly. The PRI, PW, RF, and DOA parameters were realized by controlling the DOA with phased-array technology, which further enhanced the anti-sorting ability for the signals. After analyzing the simulation results, we obtained the following conclusions. Firstly, the proposed anti-clustering method with a 3D pseudo-center wide-agility joint design and the anti-SDIF sorting method with an interval length greater than 20 times the minimum interval could guide the design of the anti-sorting signal. Secondly, compared to the classical 1D logistic, 2D Henon, and 3D Lorenz chaotic mappings, the 2D interleaving feedback hyperchaotic system designed in this paper had better complexity and stronger randomness. The 2D hyperchaotic system designed enters the chaotic state quickly and is not controlled by fractal coefficients. Finally, the designed signal could realize anti-clustering sorting and anti-SDIF sorting. Compared with the PRI random jitter signal, the PRI signal modulated by the designed hyperchaotic system showed better anti-SDIF sorting performance.

## Figures and Tables

**Table 1:** The SDIF algorithm.

Input: TOA Number of pulses *n* Threshold function *η*
Initialization: Histogram series *c*
Output: PRI, radar pulse sequence
for *j* = 1:*c*
if *n* < 5
for *i* = 1:n
$T_{i}={\mathrm{TOA}}_{i+c}-{\mathrm{TOA}}_{i}$
$\mathrm{output}\;\mathrm{PRI}\;T_{i}$
end for
if *n* > 5
for *i* = 1:n-c
$T_{i}={\mathrm{TOA}}_{i+c}-{\mathrm{TOA}}_{i}$
end for
$\mathrm{count}\;T_{i}$$\;\mathrm{to}\;\mathrm{get}\;\mathrm{the}\;\mathrm{sum}\;\mathrm{of}\;\mathrm{the}\;\mathrm{pulse}\;S_{i}$ and form a histogram
$\mathrm{if}\;S_{i}>\eta$
$\mathrm{output}\;\mathrm{PRI}\;T_{i}$ and signal pulse
end if
end for

**Table 2:** Signal simulation parameter settings.

Serial Number	Signal Parameter	Interval Central Value with Wide Agility			Interval Central Value without Wide Agility		
		Central Value	Interval	Interval Length	Central Value	Interval	Interval Length
1	PW	15 μs	[13 μs, 17 μs]	4 μs	15 μs	[13 μs, 17 μs]	4 μs
2		20 μs	[18 μs, 22 μs]	4 μs	18 μs	[16 μs, 20 μs]	4 μs
3		25 μs	[23 μs, 27 μs]	4 μs	20 μs	[18 μs, 22 μs]	4 μs
4	DOA	40°	[39.5°, 40.5°]	1°	40°	[39.5°, 40.5°]	1°
5		42°	[41.5°, 42.5°]	1°	41°	[40.5°, 41.5°]	1°
6		44°	[43.5°, 44.5°]	1°	41.5°	[41°, 42°]	1°
7	RF	3.5 GHz	[3.25 GHz, 3.75 GHz]	0.5 GHz	3.5 GHz	[3.25 GHz, 3.75 GHz]	0.5 GHz
8		4.1 GHz	[3.85 GHz, 4.35 GHz]	0.5 GHz	3.9 GHz	[3.65 GHz, 4.15 GHz]	0.5 GHz
9		4.7 GHz	[4.45 GHz, 4.95 GHz]	0.5 GHz	4.3 GHz	[4.05 GHz, 4.55 GHz]	0.5 GHz

**Table 3:** The approximate entropy values for the four different chaotic mappings varying with the fractal coefficient.

r	Logistic	Henon	Lorenz	Proposed
0.5	7.2584 × 10^−7^	−5.0011 × 10^−9^	0.3364	2.0056
1	2.0764 × 10^−5^	−1.5005 × 10^−8^	0.3409	2.0241
1.4	7.1890 × 10^−7^	0.9317	0.3233	2.0042
2	1.8422 × 10^−7^	None	0.2899	1.9907
2.5	8.2893 × 10^−7^	None	0.2760	1.9875
3	0.0111	None	0.2831	1.9708
3.5	0.0016	None	0.2815	1.9668
4	0.70	None	0.2847	1.9497

**Table 4:** Recursive quantitative analysis of different sequences.

Sequence Types	RR	DET	$L_{\max}$	ENTR
sin(2πt)	0.0918	0.9999	3141	0.2284
White gaussian noise	0.0562	0.1083	5	2.6582
Logistic	0.0850	0.6926	26	0.3611
Henon	0.0743	0.4795	24	0.3990
Lorenz	0.0685	0.2947	10	0.7926
Proposed chaotic mapping (X dimension)	0.0644	0.1737	6	1.5483
Proposed chaotic mapping (Y dimension)	0.0664	0.1900	5	1.4514

**Table 5:** The NIST-800-22 test results for the chaotic mapping designed in the paper.

Type of Test	*p*-Value (X Dimension)	*p*-Value (Y Dimension)	Result
Frequency test	0.5962	0.5589	Success
Frequency test within a block	0.6852	0.5203	Success
Runs test	0.3586	0.4562	Success
Test for the longest Rn in a block	0.9910	0.8564	Success
Binary matrix rank test	0.1925	0.1267	Success
Discrete Fourier-transform test	0.3526	0.4013	Success
Non-overlapping template matching test	0.5199	0.4663	Success
Overlapping template matching test	0.9620	0.9581	Success
Maurer’s “universal statistical” test	0.8625	0.8762	Success
Linear complexity test	0.4961	0.5019	Success
Serial test	0.1529	0.1973	Success
Approximate entropy test	0.5238	0.6801	Success
Cumulative sums test	0.8647	0.7361	Success
Random excursion test	0.4938	0.5019	Success
Random excursion variant test	0.5397	0.4963	Success

**Table 6:** The time taken by different chaotic mappings to generate sequences.

Types of Chaotic Mapping	Logistic	Henon	Proposed Chaotic Mapping
Time	0.09832	0.06719	0.1092

**Table 7:** Signal simulation parameter settings.

Serial Number	Signal Parameter	Center Value	Interval	Tolerance	The Minimum Interval between Adjacent Pulses
1	PRI	1 ms	[500 μs, 1500 μs]	200 μs	100 μs
2	RF	2 GHz	[1.75 GHz, 2.25 GHz]	100 MHz	50 MHz

## Data Availability

I choose to exclude this statement because the study did not report any data in the Data Availability Statement.
